# Multiple Cycle Slip Detection Algorithm for a Single Frequency Receiver

**DOI:** 10.3390/s22072525

**Published:** 2022-03-25

**Authors:** Young-Min Yoon, Byung-Seok Lee, Moon-Beom Heo

**Affiliations:** 1 Korea Aerospace Research Institute, University of Science and Technology, Daejeon 34133, Korea; 2 SBAS Program Office, Korea Aerospace Research Institute, Daejeon 34133, Korea; bslee@kari.re.kr; 3 GNSS R&D Division, Korea Aerospace Research Institute, Daejeon 34133, Korea; hmb@kari.re.kr

**Keywords:** cycle slip, GPS, cross-ratio

## Abstract

A satellite navigation system makes it simple to find and navigate to a specific position. Although a carrier measurement is required to establish a precise position due to the characteristics of the carrier observation, it is difficult to determine a robust position in a poor signal reception environment such as urban areas. Various studies are being carried out to overcome this problem, with cycle slips being the most important factor. With only a single frequency, it is very challenging to detect cycle slips in multiple satellite channels at the same time. A geometry-based technique is proposed in this study as a technical solution for detecting simultaneous cycle slips for multiple channels utilizing only a single-frequency receiver. The method could detect a half-wavelength size of cycle slip for each channel through the geometry information.

## 1. Introduction

GPS signal carrier phase measurements can be used to obtain high-precision positioning and navigation solutions. Measurements of the carrier phase, on the other hand, require the resolution of integer ambiguities. As long as the GPS receiver remains locked to the satellite signal, it can keep track of an integer number of cycles. In practice, several distracting variables may temporarily disrupt the GPS signal, resulting in a cycle slip (CS) in the observed carrier phase. Signal interference from obstacles, a low signal intensity, and receiver signal processing failure are all causes of CSs. If cycle slips occur, either the ambiguities must be resolved, or the cycle slips must be repaired in order to resume the accurate positioning and navigation procedure.

To avoid the delays and computational complexity associated with integer ambiguity resolution, CSs should be detected and repaired. In fact, detecting CSs from carrier phase measurements is challenging because it requires more information such as knowing the position or calculating a precise positioning beforehand. Several studies have proposed a combination of various instruments or methods for detecting and reducing CSs, but there are limitations in adverse conditions such as urban surroundings. Carrier phase measurements provide a precise position, but they necessitate the use of a dual-frequency receiver. Furthermore, in some unusual simultaneous cycle slip combinations on L1 and L2, the residual term in this combination may not provide any information about which phase the cycle slip occurred in, or it may completely miss the detection of the slip [1,2,3,4,5,6,7,8]. Although the time difference method removes ambiguous integers, it is only suitable for static positioning applications [2,9,10,11,12,13,14,15,16]. Combinations of code phase measurements are straightforward to implement, but noisy; thus, they are only used to detect large cycle slips [13,17,18,19,20]. Cycle slips are unaffected by Doppler integration techniques, but measurement error is caused by the receiver’s oscillator clock variance. High receiver dynamics also have a significant impact on them [2,9,19,21,22,23,24,25]. The receiver autonomous integrity monitoring (RAIM) methodology used in aviation is an example of a consistency check tool that uses duplicated measurements. Overlapping observations are used to verify statistical consistency; however, detecting CSs in multiple channels is difficult [12,26,27,28]. The RANCO (Range Consensus) technique solves this difficulty by calculating all possible combinations to produce a normal channel, making real-time processing on the receiver challenging [29,30,31].

The purpose of this study was to research methods for detecting CSs using only a single frequency. In an urban environment with poor satellite navigation signal reception, the following limitations are required for accurate positioning:

- The precise position based on the carrier phase measurement must be computed.
- A low-cost receiver that does not require any additional equipment needs to be used, and there will only be a single frequency available.
- CSs that appear in multiple channels should be observed at the same time.

This study proposes that a channel-specific technique must be used for multi-channel detection. In this work, we employed the ratio to detect CSs for each channel. The suggested method, which compares the ratio with the time and range domains, may identify small-sized CSs in several channels simultaneously.

## 2. Modeling

### 2.1. CS Detection Methodology

In an urban area, it is difficult to detect a CS by using a single-frequency receiver because the position is uncertain, necessitating a consistency check or position estimation. In general, it is vital to evaluate what to compare against and how to compare in order to recognize an outlier. A position domain or a time domain classification can be applied to the comparison target. Specific positions are compared, or a rate of change over time is targeted. A consistency test that compares multiple items against one and a method that compares estimations and observations are two types of comparison procedures. Detection is achieved by dividing the position-based domain and the range-based domain in the field of satellite navigation, which determines positions using triangulation utilizing measurements.

To detect a CS in each channel independently, detection should be conducted in a range-based domain rather than a position-based comparison target. The difficulty is that a comparison target, such as the consistency technique, is necessary to detect the existence or absence of an anomaly in the channel, whereas abrupt changes can be detected using the signal ratio [32]. Setting up a threshold as a judgement criterion is necessary to detect a ratio for each channel, and an invariant intersection (cross-)ratio can be a criterion in the projective transformation of the geometry [33]. The invariance of the cross-ratio may indicate that all channels have the same ratio and could also be used to create a threshold based on the size of the CS.

Models for analytic solutions and the cross-ratio are explained in this study. The cross-ratio is then used to examine the performance in the detection of CSs. In addition, the robustness of the suggested CS detection approach is proven using the multi-channel detection method by focusing on the possible scenarios.

### 2.2. GPS Observation Model

The following equations are used to represent carrier phase measurements for single, double, and triple differentials. An observation model of carrier phase measurements can be found in Equation (1) below.

(1) $$\phi \left(t\right)=r\left(t\right)+c\left[\delta t_{r}\left(t\right)-\delta t_{s}\left(t\right)\right]-I\left(t\right)+T\left(t\right)+\lambda N+\epsilon_{\phi }$$

where ϕ(=λϕ) is the measured phase between the satellite and the receiver (m); λ is the wavelength of the carrier signal; N is the integer ambiguity term; r is the true range between the satellite and the receiver (m); c is the speed of light (m/s); $\delta t_{r}\left(t\right)$ is the receiver clock bias (sec); $\delta t_{s}\left(t\right)$ is the satellite clock bias (sec); I is the ionospheric delay (m); T is the tropospheric delay (m); $\epsilon_{\phi }$ represents the receiver measurement noise and multipath or modeling errors (m).

The Doppler measurement has the following relationship (Equation (2)).

(2) $$f_{d}=-\frac{\dot{r}}{\lambda }$$

where $f_{d}$ is the observed Doppler shift; $\dot{r}$ is the line-of-sight range rate.

Using an observation model, Equations (3)–(5) are utilized to express the carrier phase with a single difference, double difference, and triple difference.

(3) $$\Delta \phi =\Delta r+\Delta \tau +\Delta T-\Delta I+\lambda \Delta N+\epsilon_{\Delta \phi }$$

where Δ is the single difference between two receivers; τ is equal to $c\delta t_{\Delta r}$ as the clock bias between receivers (m).

(4) $$\nabla \Delta \phi =\nabla \Delta r+\nabla \Delta T-\nabla \Delta I+\lambda \nabla \Delta N+\epsilon_{\nabla \Delta \phi }$$

where ∇ is the single difference between satellites, so ∇Δ is called the double difference.

(5) $$\delta \nabla \Delta \phi =\delta \nabla \Delta r+\delta \nabla \Delta T-\delta \nabla \Delta I+\epsilon_{\delta \nabla \Delta \phi }$$

where δ is the time difference between epochs, so δ∇Δ is called the triple difference.

The double difference is used to eliminate the satellite and receiver clock bias, and the triple difference can be used to remove the ambiguous integer N. The following equation (Equation (6)) is obtained when the observation model is transformed to the user’s position.

(6) $$\phi_{dd}\left(t\right)=e^{i}\left(t\right)\cdot \left(x_{u}\left(t\right)-x_{r}\left(t\right)\right)+T_{dd}\left(t\right)-I_{dd}\left(t\right)+\lambda N_{dd}+\epsilon_{\phi_{dd}}$$

where $\phi_{dd}$ equals ∇Δϕ as a double-differentiated carrier phase measurement; $e^{i}$ is the double-differentiated LOS (line-of-sight) vector which means that the single differential LOS between two receivers is the same, and the double differential LOS between two satellites is $e^{i}\;\left(=e^{i}-e^{reference\;sv}\right)$; $x_{u}$ is the position of the rover (user); $x_{r}$ is the position of the reference station.

To make the expression even simpler, the subscripts (${\;}_{dd}$) used later could be removed, making ϕ a double-differentiated value, and a clear expression could be used if necessary. Furthermore, the ambiguous integer expressed in the following means the ambiguous integer multiplied by λ with the distance unit.

### 2.3. The Doppler and Phase Rate of Change Relationship

The following status of an instantaneous epoch is described in the observation model above, and it is intended to indicate that the change in the user’s position compared to the change in the satellite’s position has a significant impact on the measured value [34]. The following equation (Equation (7)) is produced by substituting the above Equations (4) and (6).

(7) N=e⋅x+(T−I)−ϕ+ν

where $x\left(=x_{u}-x_{r}\right)$ is the distance vector between two user positions that are observing simultaneously.

A total differential with an ambiguous integer is the following in Equation (7).

(8) CS=dN=e⋅Δx+Δe⋅x+Δ(T−I)−Δϕ+Δϵ

where $e_{k-1}=e_{k}-\Delta e_{k}$ is expressed in units of the satellite change rate $\Delta e_{k}$ between two consecutive epochs.

If a CS occurs at moment k, the CS can be represented as stated in Equation (9).

(9) $$\begin{array}{c} CS_{k}=e_{k}\cdot x_{k}-e_{k-1}\cdot x_{k-1}+\Delta \left(T-I\right)-\Delta \phi +\Delta \epsilon \\ \;\;\;=e_{k}\cdot \Delta x_{k}+\Delta e_{k}\cdot x_{k-1}+\Delta \left(T-I\right)-\Delta \phi +\Delta \epsilon \end{array}$$

where $\Delta x_{k}=x_{k}-x_{k-1}$ represents the changes in the user’s position between two consecutive epochs.

Tropospheric delay is negligible when sampling at 1-s intervals under a very high level of ionospheric activities [8], and it needs an assumption that the influence of ionospheric delay is neglected by considering relative measures between consecutive epochs. If $\Delta e_{k}\cdot x_{k-1}$ is set to $\varepsilon_{k}$ in the preceding expression, it is calculated as a small change value, as shown in Equation (10) below.

(10) $$\varepsilon_{k}=\Delta e_{k}\cdot x_{k-1}\approx \frac{3.89\;\mathrm{km}/s\times 1\;s\times 10\;m}{20200\;\mathrm{km}}\approx 0.0019\;m\approx 0.01\mathrm{cycle}$$

where the approximate distance between the reference station and the satellite is 20,200 km; the satellite speed is 3.89 km/s; the pre-position error is 10 m (estimated by the code measurement); the sampling interval is 1 s; the error range is approximately 0.01 cycle.

It can be theoretically approximated with an observation error of a 0.02 cycle size, as shown above [35].

(11) $$\begin{array}{ll} CS_{k} & =e_{k}\cdot \Delta x_{k}+\varepsilon_{k}-\Delta \phi +\Delta \epsilon \\ & =0\;\left(\mathrm{if}\;\mathrm{no}\;\mathrm{CS}\right) \end{array}$$

The value caused by the change rate of the carrier phase is estimated entirely as a change in the user’s position by treating the value caused by the satellite change as noise with a size of 0.02 cycle, as shown in Equation (11).

The rate of change in $\dot{r}$ stated above is expressed as the rate of change in the observed carrier phase between two consecutive epochs in the model equation for the relationship between the measured carrier phase and Doppler, which is shown in Equation (12) below [18].

(12) $$f_{d}=\frac{\dot{\phi }}{\lambda }\to \dot{\phi }=\lambda f_{d}$$

The carrier phase measurement can be determined by integrating the Doppler measurement at each moment, as indicated in Equation (13).

(13) $${\hat{\phi }}_{t}=\lambda \int_{t_{0}}^{t}f_{d}dt$$

The following are the steps for estimating the measured phase using discrete Doppler measurements (14) [36].

(14) $${\hat{\phi }}_{k+1}=\phi_{k}+\frac{{\dot{\phi }}_{k+1}+{\dot{\phi }}_{k}}{2}dt,dN_{k+1}={\hat{\phi }}_{k+1}-\phi_{k+1}$$

The difference may be monitored from the time-differentiated phase by using the Doppler characteristic that is insensitive to CSs, as indicated in Equation (14) above, and the occurrence of CSs can be validated by setting the Doppler as an average as the estimation of the phase rate changes [9]. Since the carrier predicts a value with a comparison method through Doppler measurement, as shown in Equation (14), the rate of change occurs when a CS occurs, as shown in Equation (11), through the difference between the carrier phase measurement value change and the average Doppler measurement value. However, depending on the receiver and dynamic state, the Doppler measurement has considerable distortion and noise, necessitating a proper solution [22]. The Doppler measurement varies substantially depending on the signal quality, such as the satellite elevation and C/N0, and varies greatly from receiver to receiver. If the average Doppler value is adopted, the weights for each altitude and receiver must be calculated and added to the threshold, as shown in Equation (14). The least squares (LS) method is used to determine whether or not a CS is present by comparing the residuals between channels. When the LS turns dynamically, the calculation becomes complicated due to the statistical character of the LS, as the residuals change even for the same channel [34].

To eliminate the Doppler error and noise, a Doppler measurement was utilized with a double-differentiated carrier phase and a moving average filter to remove the noise. The carrier phase measurement using the double difference, in particular, reduced errors considerably. In the case of Doppler measurements, the receiver clock bias was removed by using the double difference, and the measurement noise was decreased by using the moving average filter under dynamic conditions.

### 2.4. Cross-Ratio CS Detection Technique

When one plane is projected onto another, a transformation relationship between the projected matching points is established, and this transformation relationship is known as homography. Planar homography in computer vision refers to the ability to translate points on a plane into a homography relationship between the points collected by each camera. Satellite navigation receivers may benefit from the same geometry used in computer vision. Because they are distant compared to the distance between both the receivers in the case of a single carrier phase difference, GPS satellites may be considered as points on a plane. The transformation relationship between the image points obtained by the two cameras may be treated as a single differential phase measurement, and each receiver can be compared to the camera taking points on the plane. Planar homography is the transformation relationship between identical points captured by two cameras, and it contains projective geometry properties.

$$\mathrm{Cross}\left({\overset{ˉ}{x}}_{1},{\overset{ˉ}{x}}_{2},{\overset{ˉ}{x}}_{3},{\overset{ˉ}{x}}_{4}\right)=\frac{\left|{\overset{ˉ}{x}}_{1}{\overset{ˉ}{x}}_{2}\right|\left|{\overset{ˉ}{x}}_{3}{\overset{ˉ}{x}}_{4}\right|}{\left|{\overset{ˉ}{x}}_{1}{\overset{ˉ}{x}}_{3}\right|\left|{\overset{ˉ}{x}}_{2}{\overset{ˉ}{x}}_{4}\right|}$$

where $\left|{\overset{ˉ}{x}}_{i}{\overset{ˉ}{x}}_{j}\right|=\det\left[\begin{array}{c} x_{i1}\;\;\;\;x_{j1}\\ x_{i2}\;\;\;\;x_{j2} \end{array}\right]$ is the distance between two points in one dimension as a determinant; ${\overset{ˉ}{x}}_{i,j}$ is a point in the homogeneous coordinates.

**Figure 1:**
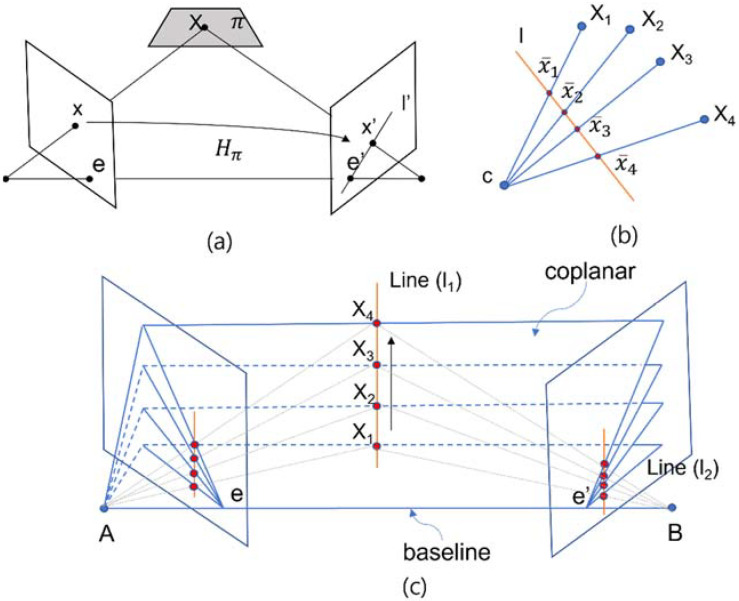
Projective geometry and homogeneous coordinates. (**a**) A point x is transferred via the plane π to a matching point $x^{\prime }$. (**b**) Concurrent lines. (**c**) As the position of the 3D point $X_{1},\ldots ,X_{4}$ varies, the epipolar planes rotate about the baseline.

Hartley and Zisserman (2003, 259) stated that the epipolar line is the projection in the second image of the ray from the point x through the camera center C of the first camera. Thus, there is a map $x\mapsto l^{\prime }$ from a point in one image to its corresponding epipolar line in the other image, which derives the homogeneous representative. The ray corresponding to a point x is extended to meet the plane π in a point $X_{\pi }$ in Figure 1a. Hartley and Zisserman (2003, 259) defined epipolar lines as follows: “An epipolar line is the intersection of an epipolar plane with the image plane. All epipolar lines intersect at the epipole. An epipolar plane intersects the left and right image planes in epipolar lines, and defines the correspondence between the lines”. As scholars have pointed out, a geometric derivation of corresponding points:

Consider a plane π in space not passing through either of the two camera centres. The ray through the first camera centre corresponding to the point meets the plane π in a point X. This point X is then projected to a point $x^{\prime }$ in the second image. This procedure is known as transfer via the plane π. Since X lies on the ray corresponding to x, the projected point $x^{\prime }$ must lie on the epipolar line $l^{\prime }$ corresponding to the image of this ray …. The points and are both images of the 3D point X lying on a plane. The set of all such points $x_{i}$ in the first image and the corresponding points $x_{i}^{\prime }$ in the second image are projectively equivalent, since they are each projectively equivalent to the planar point set $X_{i}$. Thus there is a 2D homography $H_{\pi }$ mapping each $x_{i}$ to $x_{i}^{\prime }$.

(Hartley and Zisserman, 2003, 261)

In Figure 1b, the line l is in the time domain. Points such as $X_{1},\ldots ,X_{4}$ represent the single differential receiver, and point c describes the satellite. Note how Figure 1b may be thought of as representing a projection of points in $P_{2}$ into a 1D image:

If c represents a camera centre, and the line represents an image line (1D analogue of the image plane), then the points are the ${\bar{x}}_{i}$ projections of points $X_{i}$ into the image. The cross-ratio of the points ${\bar{x}}_{i}$ characterizes the projective configuration of the four image points. Note that the actual position of the image line is irrelevant as far as the projective configuration of the four image points is concerned—different choices of image line give rise to projectively equivalent configurations of image points.

(Hartley and Zisserman, 2003, 64)

Due to the similarity between the epipolar view and differential satellite navigation, we can make the following replacements in Figure 1c:

- Single differential case: points A and B are receivers, and $X_{i}$ is a satellite;
- Double differential case: points A and B are satellites, and $X_{i}$ is a single differential receiver;
- Triple differential case: $X_{i}$ is acquired sequentially from the double differential receiver’s measurement.

In Figure 1a, lines $l_{1}$ and $l_{2}$ are in a relationship as a projective transformation. Therefore, we can apply the cross-ratio to the CS per channel through the concept of projective mapping. In Figure 1b,c, each point ${\bar{x}}_{i}$ in the homogeneous coordinates ${\left(x_{1},x_{2}\right)}^{T}$ is a finite point, and the homogeneous representative is chosen such that $x_{2}=1,\ldots ,4$ (time or epoch); therefore, $\left|{\bar{x}}_{i}{\bar{x}}_{j}\right|$ represents the signed double differential phase measurement on each epoch. The homography between corresponding lines $l_{1}↔l_{2}$ is induced by the projection of points. Additionally, Hartley and Zisserman stated, “A 1D image is formed by the intersection of the rays $l_{i}=\bar{cx_{i}}$ with the image line l. The set of image points $\left\{{\overset{ˉ}{x}}_{i}\right\}$ is projectively equivalent to the set of rays $\left\{l_{i}\right\}$. For four points, the projective equivalence class of the image is determined by the cross-ratio of the points” (Hartley and Zisserman, 2003, 553).

At the same epoch, the difference between the double differential carrier phase measurement and the Doppler measurement is projected onto the baseline in Figure 1c. We can acquire the cross-ratio of points, which is the triple differential observation value in each channel.

This method finds the cross-ratio of each channel, and therefore it does not require any estimation or redundancy. An individual CS for each channel can be detected, and the comparison check for each channel can be performed using the consistency check via the ratio. Furthermore, due to the application of the geometric concept, the detectable CS size is determined by the signal quality rather than the physical distance between the receivers.

## 3. Cross-Ratio CS Detection Performance

In order to use the concept of the cross-ratio for CS detection, an input needs to be chosen first and then the threshold’s properties need to be examined. Because the cross-ratio is a geometric concept, it must be satisfied regardless of the base distance between the two receivers being used. In this experiment, for example, a comparatively long distance (22.5 km) was used in the differential phase measurement between receivers.

First, the measured phase estimates and the residual were examined with respect to the input in a distinctive manner. In terms of threshold setting, a study was carried out to examine whether it was fixed or not, as well as the size of the detectable CS. The cross-ratio of single, double, and triple differential phase measurements is the same for each measurement, according to the analysis results. All the results for the measured phase, estimate, and residual are 0.25, and the measured phase can be used to detect the half-wavelength size of CSs.

### 3.1. Analyzing Characteristics Based on Input

The results of the cross-ratio for each measurement (single, double, and triple differential) as input are shown in Table 1 below, with all cross-ratios having a value of 0.25.

**Figure 2:**
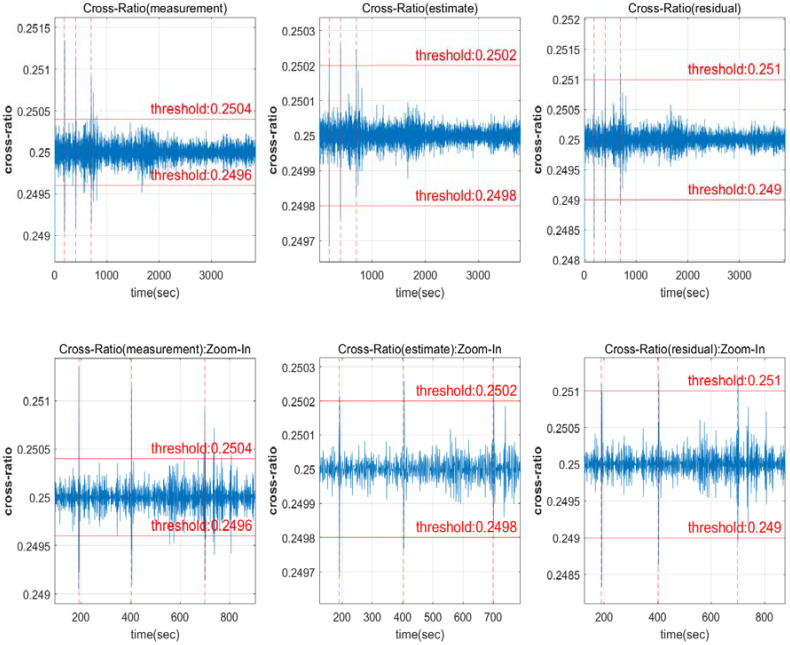
Satellite 13 input value comparison ((**lower row**) is a zoomed view of the (**upper row**)).

The results of the measured phase, estimated phase, and residual cross-ratio are shown in Figure 2. The value of the cross-ratio remains identical as 0.25 for each measurement result. However, as the signal noise in the residual is low, an accurate threshold setting is achievable.

A Kalman filter was employed in the estimate of Figure 2 for the input comparison. In terms of the residual magnitude, a result was obtained that is capable of detecting half-wavelength CSs. The required size could be provided for manually setting the threshold. However, depending on the signal quality, the properties may change, necessitating calculation for the estimation. It is simple to compute using the measurements, but in order to avoid false alarms caused by noise in the measurements, the threshold of a specific magnitude or more must be specified.

**Figure 3:**
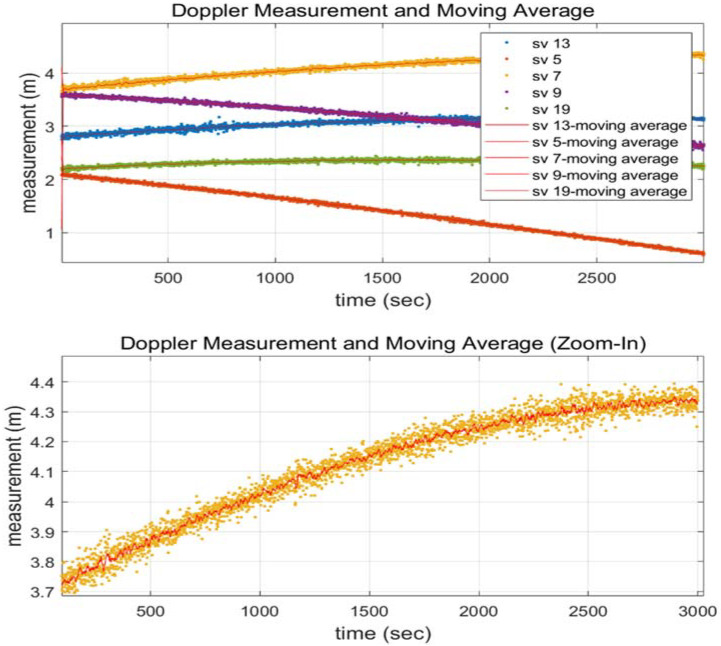
Each Doppler measurement’s moving average ((**lower row**) is a zoomed view of the (**upper row**)).

**Figure 4:**
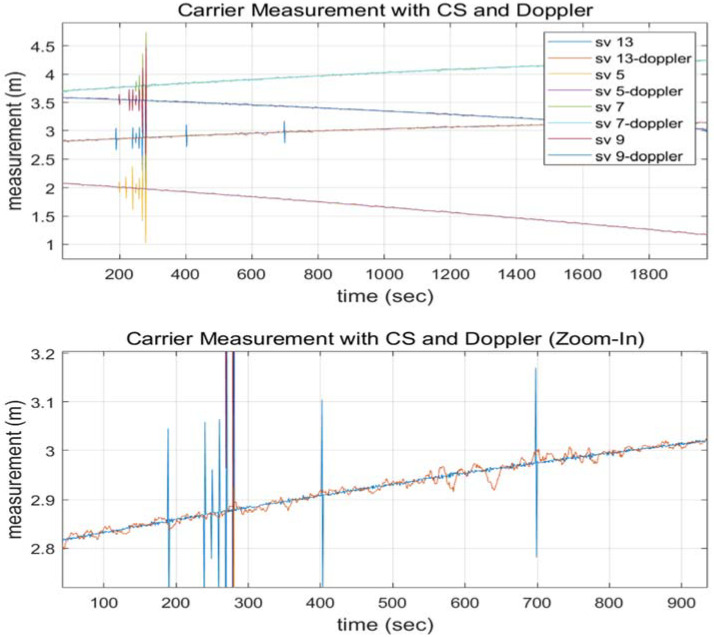
Doppler moving average and carrier measurements with a CS ((**lower row**) is a zoomed view of the (**upper row**)).

The main source of noise when employing carrier phase and Doppler measurements is called Doppler. A moving average value can be utilized instead of the Doppler average between two successive epochs to produce a better quality measurement. Figure 3 shows that the 10-s interval moving average filter estimate (red line in the below plot) outperforms the 2-s interval average filter estimate (yellow dots in the below plot). The carrier measurements with a CS and the moving averaged Doppler are shown in Figure 4 to validate that the two signals are matched without bias. Noise-removed Doppler measurements were used to examine this paper.

### 3.2. Determination of the Threshold

These are the findings of an investigation of whether the threshold is set and fixed in relation to the CS size, which is a factor to consider when determining the threshold.

**Figure 5:**
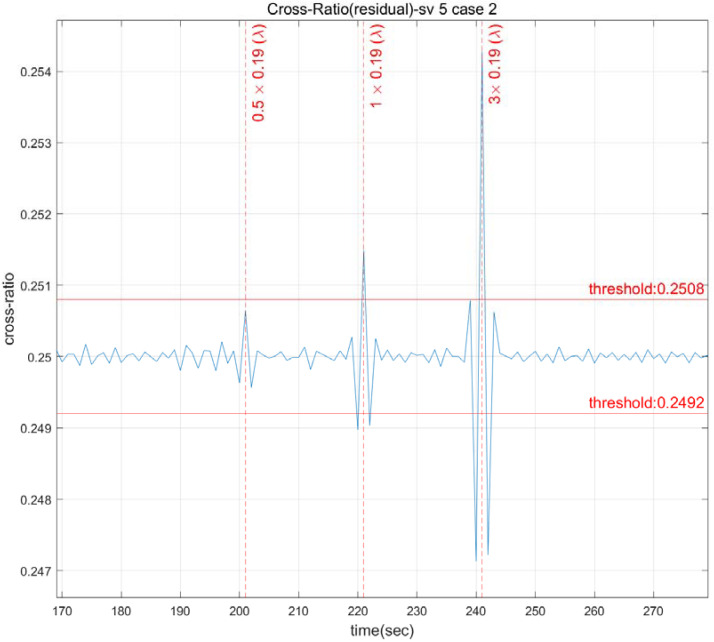
The cross-ratio as a function of the CS size.

Because of the nature of the cross-ratio, quantitative analysis is required due to qualitative analysis being problematic when defining the threshold. Specifically, the threshold size is decided by the correlation between the CS size and the signal noise despite the fact that the cross-ratio is the same regardless of the input. The cross-ratio by CS size is shown in Figure 5. The CS detection threshold in Figure 5 is an arbitrarily chosen value for each channel. The cross-ratio of one wavelength size for CS detection is 0.25149 in the measurement of satellite 5, and the cross-ratio of three wavelength sizes is 0.25428, demonstrating that the ratio value grows as the CS size increases.

The cross-ratio is a correlation between the signal noise level and the detectable threshold, where the more the signal noise increases, the larger the detectable threshold becomes. Figure 2 illustrates how the detectable threshold varies based on the measurement and estimates of both thresholds, which are set at 0.2504 and 0.2502, respectively. To fix this, either signal noise must be reduced, or the detection threshold must be increased. It is necessary to determine the threshold that can be fixed regardless of the channel.

**Figure 6:**
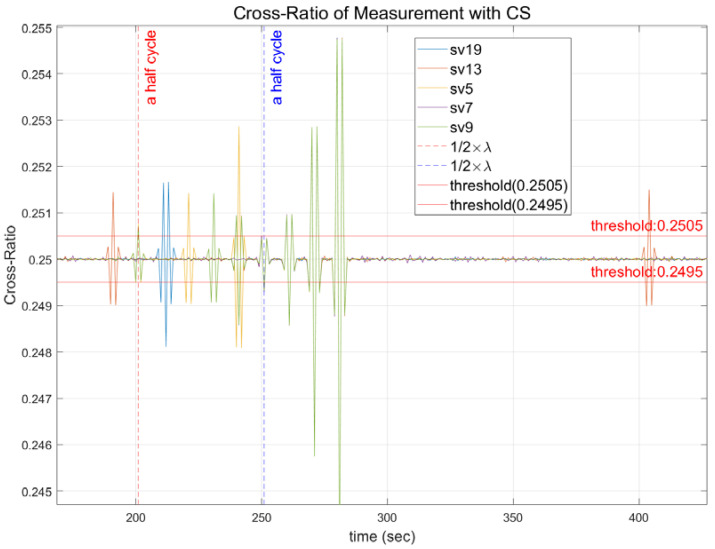
Determination of the half-wavelength size threshold.

Figure 6 shows the outcome of the Doppler measurement after adopting the moving average filter to eliminate noise from the measurement. The cross-ratio of the noise-removed signal can be set to a half-wavelength threshold in all channels, as shown in Figure 6. The specified thresholds have 0.2505 and 0.2495 as the upper and lower bounds, respectively.

## 4. CS Detection Technique with Multiple Channels

The CS detection must separate the originating channel from the reference satellite or multiple channels because this article uses the double differential measurement. In general, the size and direction of the generated CS are used to determine whether the CS occurred in the reference satellite or in each channel due to the nature of the cross-ratio. However, if multiple CSs occur at the same time with the same size, different scenarios appear, necessitating verification. Experiments were carried out by separating the scenario composition into two categories: a case where the CS size is not the same, and a case where it does not matter.

### 4.1. Scenarios of Non-Equal CS Size Detection

Cases can be classified into two categories based on the CS detection state: cases where a CS occurs in all channels, and cases where a CS occurs only partially. This can ascertain whether CS sizes are the same or not.

The scenario for detecting non-identical CS sizes was built on the assumption that the same CS size does not occur at the same time. This is a scenario in which a CS is detected in all channels and can be categorized based on the size of the CS and the direction in which it occurs. The scenario was set up as shown in the Table 2 below, where ○ (all) means that a CS occurs in the reference satellite or all channels, and ⅹ (no) means no CS occurs.

When they occur in the reference satellite or all channels, all scenarios, “1-1” to “1-3”, are feasible and can be identified by CS properties. Overall, the condition of CS detection can be characterized by whether a CS happens in the reference satellite or in all channels except the reference satellite. The CS detection state appears as a result of occurrence in all channels in scenario “1-1”. Scenario “1-2” has a CS in all channels except the reference satellite, while scenario “1-3” has a CS in the reference satellite and all channels at the same time. Table 3 below shows the artificial CS size from scenario “1-1” to “1-3”.

**Figure 7:**
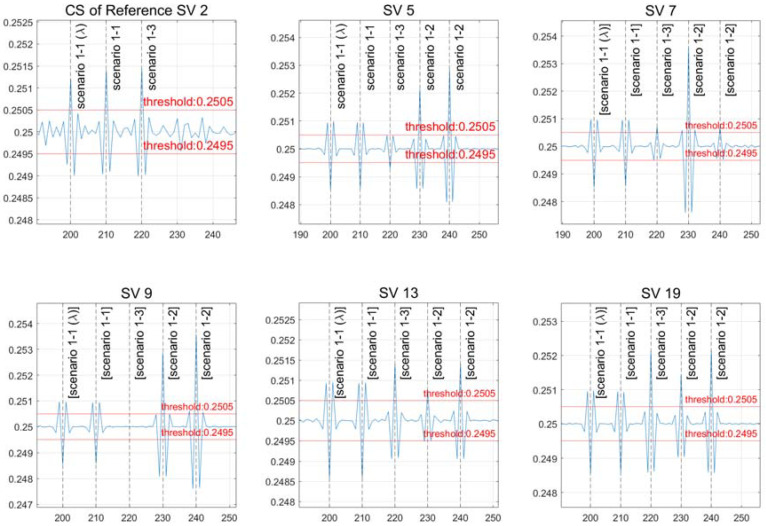
The outcomes of a scenario with different CS sizes.

The experimental results for each case are displayed in Figure 7 below after inputting an artificial CS, as shown in the table above.

Scenario “1-1” describes a scenario in which all detected CS sizes are the same and are exceeded in the same direction (indicating the same threshold). Scenario “1-1” shows that a CS is formed in all channels with the same magnitude and direction, whereas a CS is generated just for the reference satellite at 200 and 210 s.

At 230 and 240 s in scenario “1-2”, CSs of different sizes can be detected. Except for satellite 9, a CS occurs in all channels and reference satellites at 220 s in scenario “1-3”, and a CS can be recognized in all directions and sizes. The ninth satellite is a case in which the CS size is the same, and the elements of this scenario are discussed in Section 4.2 “Scenarios of Equal CS Size Detection”.

### 4.2. Scenarios of Equal CS Size Detection

The scenarios can be designed as shown in Table 4 below, in which a CS is partially detected, rather than the assumption that the CS sizes are not the same as above. △ (partial) denotes the presence of a CS but not its detection in Table 4.

The scenario described above is one in which no assumptions about the CS size are made, and in which CS detection occurs in all channels. In the worst-case scenario, a CS may be detected in only a portion of the channel, or it may not be detected at all.

The meaning of the scenario number is to distinguish it from the assumption that a CS occurs in all or partial channels in the case of “2”, and the meanings of “A” to “C” are to distinguish whether a CS occurs in the reference satellite and whether a CS occurs in all or partial channels in the case of “1”. “A” indicates that a CS occurs in the reference satellite and all channels, while “B” indicates that a CS occurs in the reference satellite but only in some channels. Additionally, “C” denotes a scenario in which a CS occurs only in a subset of channels. After letters “A” through “C”, the meanings of “0” and “1” show if the CS sizes are equal or not. CSs of the same size occur simultaneously in the reference satellite and all channels in scenario “2-A0”, while CSs of different sizes occur in the same condition in scenario “2-A1”.

**Figure 8:**
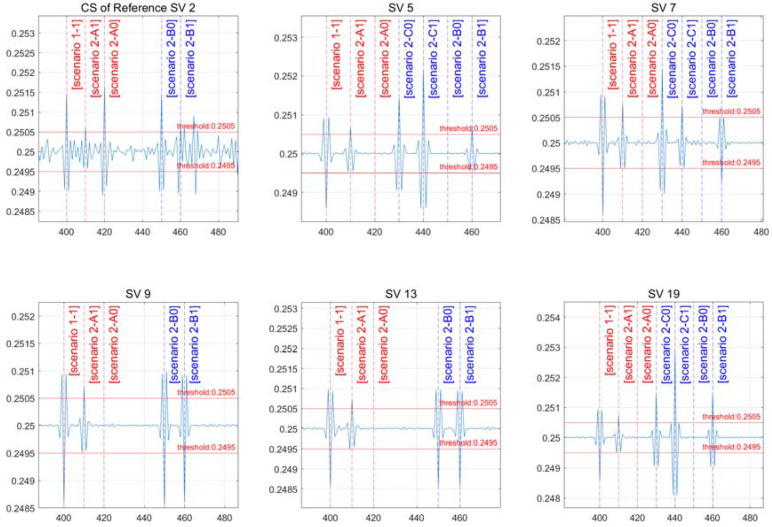
Results of a scenario with the same CS size.

Table 5 shows the artificial CS size and time for implementing the scenario, with the result presented in Figure 8.

Figure 8 shows whether a CS has occurred in the reference satellite, and the remaining figures indicate the status of CS incidence for each satellite. For each epoch, a CS is generated based on the scenario. At 400 s, scenario “1-1” occurs; at 410 s, scenario “2-A1” occurs; and at 420 s, scenario “2-A0” occurs. A CS was created exclusively in satellites 5, 7, and 19 at 430 and 440 s in scenarios “2-C0” and “2-C1” that occur only in some channels. Scenarios “2-B0” and “2-B1” were set to 450 and 460 s, respectively, in which a CS occurs only in the reference satellite and some channels.

Figure 8 shows the CS occurrence of reference satellite 2, which is shown in the top left figure as a single differential to indicate the CS occurrence for each scenario. CSs of one wavelength, half a wavelength, and one wavelength occur at 400, 410, and 420 s in scenarios “1-1”, “2-A1”, and “2-A0”. Scenarios “2-B0” and “2-B1” happen at 450 and 460 s, respectively, with one wavelength and half a wavelength.

Figure 8 depicts scenarios “2-A0” and “2-A1” in which CSs occur concurrently in all channels. A CS is not detected in all channels when “2-A0” has the same CS size. For each satellite in scenario “2-A0”, a CS of the same size happens at 420 s in the reference satellite and all channels, canceling out CS detection. In scenario “2-A1”, the CS size differs between the half-wavelength reference satellite channel and the other one-wavelength channel, resulting in all channels being identified with the same half-wavelength size.

Scenarios “2-B0” and “2-B1” depict a scenario where the reference satellite and some channels are susceptible to CSs. Instead of the channels where the CS does not occur, the CS is not detected in the channel where it occurs. CS detection happened in channels 9 and 13 of the reference satellite in scenario “2-B0”, and the detection resulted in unexpected conclusions. In some channels, satellites 5, 7, and 19, and the reference satellite, scenarios “2-B0” and “2-B1” occur at 450 and 460 s. This is a condition in which the reference satellite’s CS detects a CS in channels 9 and 13. This occurs when the CS is not detected in the channel where it is formed, while the reverse is detected in the channel where it does not occur. Even if a CS is identified, exclusion or mitigation should not be performed unconditionally. Even though the CS is not a usual detected circumstance, this demands additional confirmation work.

Scenarios “2-C0” and “2-C1” describe a scenario in which a CS is not formed in the reference satellite but occurs in specific channels and is detected exclusively in those channels.

The scenario findings in Table 6 below show that it is possible to detect a CS in channels where the CS does not occur, or not to detect a CS in channels where the CS occurs. For each channel, an additional validation method for CS detection is necessary. The cross-ratio of a single differential measurement can be used as a supplementary detection approach. The threshold setting in the case of a single differential may be larger than in the case of a double differential, but as the following experimental findings show, the threshold setting is unimportant because it is simply used to check only if a CS is detected.

### 4.3. The Same CS Size Detection Method

**Figure 9:**
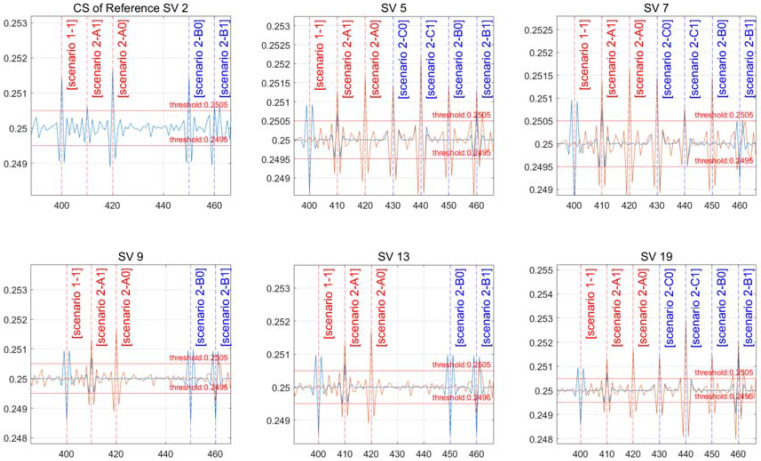
Single/double differential measurement cross-ratio and CS detection scenario results.

Figure 9 shows the cross-ratios from Figure 8 and Figure 9 combined into a single differential cross-ratio value. The cross-ratio of the double differential measurement is shown by the blue line in Figure 9, while the cross-ratio of the single differential measurement is represented by the red line.

Figure 8 shows that a CS occurs in all channels of scenarios “2-A0” and “2-A1” at the same time. A CS is not detected in all channels when “2-A0” has the same CS. However, as shown in Figure 9, the cross-ratio of single differential values in all channels indicates that a CS occurred in the reference satellite and all channels. It can be confirmed that CSs of various sizes are formed in scenario “2-A1”, but it can also be verified that they occur in the reference satellite at the cross-ratio of the single differential measurement.

The circumstances of scenarios “2-B0” and “2-B1” demand extensive verification since a CS can be identified in all channels or only in channels where a CS does not occur. The cross-ratio of a single differential value validates if a CS has happened in each channel, as shown in Figure 9, allowing for unambiguous CS detection. Even if a CS is not identified, as indicated in Table 6 below, it is required to check whether a CS is detected using the cross-ratio of the single differential measurement.

When a CS is detected, the OR logic operation of the cross-ratio of the double differential and single differential is used to identify the CS occurrence channel in Table 7 below. Scenarios 2 and 1 have the following relationship, as shown in Table 7. Scenarios “2-A0” and “2-A1” are identical to scenario “1-3”, whereas scenarios “2-B0” and “2-B1” are divided into scenarios “1-1” and “1-3”, respectively. Scenarios “2-C0” and “2-C1” are equal to scenario “1-2”. Since the CS size between the reference satellite and the channel is the same in scenarios “1-3” and “2-B0”, a CS cannot be detected in the double differential (DD) carrier measurement. As a result, CS detection can be validated by a single differential (SD) measurement, which is the same as in the “2-A0” and “1-3” scenarios. Otherwise, since a CS is detected in the DD, the CS originating channel must be identified from the reference satellite or individual channels. The combination of the SD and DD can identify the CS originating channel.

In summary, the occurrence of a CS in the SD means that it occurs in the channel. Additionally, if it is not the same size as the reference satellite, it can be detected in the DD. However, continuous monitoring is required in the SD in preparation for the simultaneous occurrence of the same CS size between the reference satellite and the channel, which is the case where a CS cannot be detected in the DD.

## 5. Discussion and Conclusions

Detecting multi-channel CSs using carrier measurements is normally required for a dual-frequency receiver. The method proposed in this paper enables detecting a CS that occurs simultaneously in multiple channels at a rate that varies for each channel through a single-frequency signal. Specifically, the ratio refers to an invariant cross-ratio that remains constant even after the projective transformation, and it is possible to detect a small CS for each channel and determine whether the channels are consistent. It is possible to detect a CS with a half-wavelength size by using the algorithm provided in this work. Furthermore, it was shown that channel ambiguity, which is caused by the double differential carrier phase used to remove errors, can be identified through various scenarios. The computing power is very small because the algorithm is a simple combination of measurements; hence, it is suitable for real-time operation. This paper shows that CS detection is possible even in extreme scenarios such as in the case where the same size CS occurs simultaneously in the reference satellite and each channel. It is believed that this research method could further be expanded to the detection of multiple faults in signals as it can be used to detect outliers beyond CS detection.

## Figures and Tables

**Table 1:** Cross-ratio value of each measurement.

Measurement	ϕ	Δϕ	∇Δϕ	$\nabla \Delta \phi -f_{d}$
Cross-ratio value	0.25	0.25	0.25	0.25

**Table 2:** Scenarios of non-equal CS sizes.

Scenario	Reference sv	Each Channel	CS Detection (Size/Direction)
1-1	○ (all)	ⅹ (no)	○ (all) (same size/direction)
1-2	ⅹ (no)	○ (all)	○ (all) (different size)
1-3	○ (all)	○ (all)	○ (all)

○ (all) means that a CS occurs in all channels; ⅹ (no) means no CS occurs.

**Table 3:** Artificial non-equal-size CS input scenarios in different epochs.

**Epoch**	200	210	220	230	240
**sv 2(ref)**	λ	λ	λ	-	-
**sv 5**	-	-	$\frac{1}{2}\times \lambda$	$\frac{3}{2}\times \lambda$	$\frac{4}{2}\times \lambda$
**sv 7**	-	-	$\frac{3}{2}\times \lambda$	$\frac{5}{2}\times \lambda$	$\frac{1}{2}\times \lambda$
**sv 9**	-	-	$\frac{2}{2}\times \lambda$	$\frac{4}{2}\times \lambda$	$\frac{5}{2}\times \lambda$
**sv 13**	-	-	$\frac{4}{2}\times \lambda$	$\frac{1}{2}\times \lambda$	$\frac{2}{2}\times \lambda$
**sv 19**	-	-	$\frac{5}{2}\times \lambda$	$\frac{2}{2}\times \lambda$	$\frac{3}{2}\times \lambda$

**Table 4:** Scenarios of equal CS sizes.

Scenario	Reference sv	Each Channel	CS Detection	CS Size
2-A0	○ (all)	○ (all)	△ (partial)	same
2-B0	○ (all)	△ (partial)	△ (partial)	same
2-C0	ⅹ (no)	△ (partial)	△ (partial)	same
2-A1	○ (all)	○ (all)	△ (partial)	difference
2-B1	○ (all)	△ (partial)	△ (partial)	difference
2-C1	ⅹ (no)	△ (partial)	△ (partial)	difference

○ (all) = a CS occurs in all channels; ⅹ (no) = no CS occurs; △ (partial) = a CS partially occurs.

**Table 5:** Artificial equal-sized CS input scenarios in different epochs.

**Epoch**	400	410	420	430	440	450	460
**sv 2(ref)**	λ	$\frac{1}{2}\times \lambda$	λ	-	-	λ	λ
**sv 5**	-	λ	λ	λ	$\frac{3}{2}\times \lambda$	λ	$\frac{3}{2}\times \lambda$
**sv 7**	-	λ	λ	λ	$\frac{1}{2}\times \lambda$	λ	$\frac{1}{2}\times \lambda$
**sv 9**	-	λ	λ	-	-	-	-
**sv 13**	-	λ	λ	-	-	-	-
**sv 19**	-	λ	λ	λ	$\frac{4}{2}\times \lambda$	λ	$\frac{4}{2}\times \lambda$
**Scenario**	1-1	2-A1	2-A0	2-C0	2-C1	2-B0	2-B1

**Table 6:** Scenario analysis of equal CS size detection.

**Scenario “2-A0”**
No CS is detected in all channels (5, 7, 9, 13, 19) because the reference satellite and all channels cancel each other out due to the same-sized CS.
**Scenario “2-A1”**
A half-wavelength CS occurs in all channels (5, 7, 9, 13, 19) because of the discrepancy in the half-wavelength magnitude between the reference satellite and all channels.
**Scenario “2-B0”**
A CS is not detected in channels (5, 7, 19) because of the same CS size.
A CS is detected in channels (9, 13) due to the reference satellite’s CS.
**Scenario “2-B1”**
CS detection occurs across all channels (5, 7, 9, 13, 19), regardless of CS occurrence.
There are different CS sizes between channels (5, 7, 9) and the reference satellite.
A CS is detected in channels 9 and 13 due to the reference satellite’s CS.
**Scenario “2-C0”**
A CS is detected in channels (5, 7, 9).
**Scenario “2-C1”**
A CS is detected in channels (5, 7, 9).

**Table 7:** Combination of the SD and DD according to CS occurrence scenarios.

Scenario 2	Result			Cause (CS)			Scenario 1
	DD * Detection	SD ** Detection	Checking	Ref	Channel	CS Size	
**2-A0**	ⅹ	○	SD check	○	○	Same	1-3
**2-A1**	○	○	Identify	○	○	Diff	1-3
**2-B0**	ⅹ	○	SD check	○	○	Same	1-3
	○	ⅹ	Identify	○	ⅹ	Same	1-1
**2-B1**	○	○	Identify	○	○	Diff	1-3
	○	ⅹ	Identify	○	ⅹ	Diff	1-1
**2-C0**	○	○	Identify	ⅹ	○	Same	1-2
**2-C1**	○	○	Identify	ⅹ	○	Diff	1-2

* DD = double differential; ** SD = single differential. ○ (all) = a CS occurs in all channels; ⅹ (no) = no CS occurs.

## References

[1] Christian A. Cycle Slip Detection and Correction by Means of Integrated Systems. Proceedings of the Proceedings of the 2000 National Technical Meeting of The Institute of Navigation.

[2] Simon B., Langley R.B. (2010). Instantaneous Cycle-Slip Correction for Real-Time PPP Applications. Navig.-J. Inst. Navig..

[3] Zainab F.S., Yang D., Jin T., Echoda N. Survey of Cycle Slip Detection & Correction Techniques for Single Frequency Receivers. Proceedings of the 2018 IEEE 18th International Conference on Communication Technology (ICCT).

[4] Ji S., Chen W., Weng D., Wang Z., Ding X. (2013). A Study on Cycle Slip Detection and Correction in Case of Ionospheric Scintillation. Adv. Space Res..

[5] Kim D., Langley R.B. Instantaneous Real-Time Cycle-Slip Correction of Dual-Frequency GPS Data. Proceedings of the International Symposium on Kinematic Systems in Geodesy, Geomatics and Navigation (KIS 2001).

[6] Yukihiro K., Sone K., Sugimoto S. Cycle Slip Detection and Correction for Kinematic GPS Based on Statistical Tests of Innovation Processes. Proceedings of the 17th International Technical Meeting of the Satellite Division of The Institute of Navigation (ION GNSS 2004).

[7] Ning L., Zhang Q., Zhang S., Wu X. (2021). Algorithm for Real-Time Cycle Slip Detection and Repair for Low Elevation GPS Undifferenced Data in Different Environments. Remote Sens..

[8] Liu Z. (2011). A New Automated Cycle Slip Detection and Repair Method for a Single Dual-Frequency GPS Receiver. J. Geod..

[9] Simon B., Langley R. Cycle-Slip Correction for Single-Frequency PPP. Proceedings of the 25th International Technical Meeting of the Satellite Division of the Institute of Navigation 2012, ION GNSS 2012.

[10] Seigo F., Saito S., Yoshihara T. (2013). Cycle Slip Detection and Correction Methods with Time-Differenced Model for Single Frequency GNSS Applications. Trans. Inst. Syst. Control. Inf. Eng..

[11] Hui H., Fang L. GPS Cycle Slip Detection and Correction Based on High Order Difference and Lagrange Interpolation. Proceedings of the 2009 2nd International Conference on Power Electronics and Intelligent Transportation System (PEITS).

[12] Kirkko-Jaakkola M., Traugott J., Odijk D., Collin J., Sachs G., Holzapfel F. A Raim Approach to GNSS Outlier and Cycle Slip Detection Using L1 Carrier Phase Time-Differences. Proceedings of the 2009 IEEE Workshop on Signal Processing Systems.

[13] Lin S.-G., Yu F.-C. (2013). Cycle Slips Detection Algorithm for Low Cost Single Frequency GPS RTK Positioning. Surv. Rev..

[14] Chuang Q., Liu H., Zhang M., Shu B., Xu L., Zhang R. (2016). A Geometry-Based Cycle Slip Detection and Repair Method with Time-Differenced Carrier Phase (TDCP) for a Single Frequency Global Position System (GPS) + BeiDou Navigation Satellite System (BDS) Receiver. Sensors.

[15] Rapoport L.B. Compressive Sensing Approach for the Cycle Slips Detection, Isolation, and Correction. Proceedings of the 27th International Technical Meeting of the Satellite Division of The Institute of Navigation (ION GNSS+ 2014).

[16] Zangeneh-Nejad F., Amiri-Simkooei A.R., Sharifi M.A., Asgari J. (2017). Cycle Slip Detection and Repair of Undifferenced Single-Frequency GPS Carrier Phase Observations. GPS Solut..

[17] Liu G. (2001). Real-Time Positioning Algorithm with Single Frequency GPS Phase and Pseudo-Range and Detection of Cycle Slip. Crustal Deform. Earthq..

[18] Malek K., Karamat T., Noureldin A., El-Shafie A. (2014). GPS Cycle Slip Detection and Correction at Measurement Level. Br. J. Appl. Sci. Technol..

[19] Ren Z., Li L., Zhong J., Zhao M., Shen Y. (2011). A Real-Time Cycle-Slip Detection and Repair Method for Single Frequency GPS Receiver. Int. Proc. Comput. Sci. Inf. Technol..

[20] Tomoji T., Yasuda A. Cycle Slip Detection and Fixing by MEMS-IMU/GPS Integration for Mobile Environment RTK-GPS. Proceedings of the 21st International Technical Meeting of the Satellite Division of The Institute of Navigation (ION GNSS 2008).

[21] Mojtaba B., Ziebart M. (2010). Instantaneous Doppler-Aided RTK Positioning with Single Frequency Receivers. Proceedings of the IEEE/ION Position, Location and Navigation Symposium.

[22] Carcanague S. Real-Time Geometry-Based Cycle Slip Resolution Technique for Single-Frequency PPP and RTK. Proceedings of the 25th International Technical Meeting of the Satellite Division of The Institute of Navigation (ION GNSS 2012).

[23] Peter C., Plausinaitis D. (2014). Cycle Slip Detection in Single Frequency GPS Carrier Observations Using Expected Doppler Shift. Nord. J. Surv. Real Estate Res..

[24] Lipp A., Gu X. Cycle-Slip Detection and Repair in Integrated Navigation Systems. Proceedings of the 1994 IEEE Position, Location and Navigation Symposium-PLANS’94.

[25] Zhao J., Hernández-Pajares M., Li Z., Wang L., Yuan H. (2020). High-Rate Doppler-Aided Cycle Slip Detection and Repair Method for Low-Cost Single-Frequency Receivers. GPS Solut..

[26] Cao K., Hu Y., Xu J., Li B. Research on Improved RAIM Algorithm Based on Parity Vector Method. Proceedings of the 2013 International Conference on Information Technology and Applications.

[27] Feng S., Ochieng W., Walsh D., Ioannides R. (2006). A Measurement Domain Receiver Autonomous Integrity Monitoring Algorithm. GPS Solut..

[28] Jiang Y., Wang J. (2014). A-RAIM and R-RAIM Performance Using the Classic and MHSS Methods. J. Navig..

[29] Schroth G., Ene A., Blanch J., Walter T., Enge P. Failure Detection and Exclusion via Range Consensus. Proceedings of the European Navigation Conference 2008.

[30] Schroth G., Rippl M., Ene A., Blanch J., Belabbas B., Walter T., Enge P., Meurer M. Enhancements of the range consensus algorithm (RANCO). Proceedings of the 21st ITM of ION GNSS Conference.

[31] Zhang M., Zhang J., Zhu Y. Enhancements of the DichoTomy Based RAIM. Proceedings of the 22nd International Technical Meeting of the Satellite Division of The Institute of Navigation (ION GNSS 2009).

[32] Basseville M., Nikiforov I. (1993). Detection of Abrupt Changes: Theory and Application.

[33] Richard H., Zisserman A. (2003). Multiple View Geometry in Computer Vision.

[34] Emanuele T., Verri A. (1998). Introductory Techniques for 3-D Computer Vision.

[35] Chen Q., Chen H., Jiang W., Zhou X., Yuan P. (2018). A New Cycle Slip Detection and Repair Method for Single-Frequency GNSS Data. J. Navig..

[36] Spangenberg M., Tourneret J.-Y., Calmettes V., Duchateau G. Detection of Variance Changes and Mean Value Jumps in Measurement Noise for Multipath Mitigation in Urban Navigation. Proceedings of the 2008 42nd Asilomar Conference on Signals, Systems and Computers.

